# The Hunchback temporal transcription factor establishes, but is not required to maintain, early-born neuronal identity

**DOI:** 10.1186/s13064-017-0078-1

**Published:** 2017-01-31

**Authors:** Keiko Hirono, Minoree Kohwi, Matt Q. Clark, Ellie S. Heckscher, Chris Q. Doe

**Affiliations:** 1 Howard Hughes Medical Institute, Eugene, 97403 USA; 2 Institute of Molecular Biology, Eugene, 97403 USA; 3 Institute of Neuroscience, University of Oregon, Eugene, 97403 USA; 4 Department of Neuroscience, Columbia University Medical Center, New York, NY 10032 USA; 5 Department of Molecular Genetics and Cell Biology, University of Chicago, Chicago, IL 60637 USA

## Abstract

**Background:**

*Drosophila* and mammalian neural progenitors typically generate a diverse family of neurons in a stereotyped order. Neuronal diversity can be generated by the sequential expression of temporal transcription factors. In *Drosophila*, neural progenitors (neuroblasts) sequentially express the temporal transcription factors Hunchback (Hb), Kruppel, Pdm, and Castor. Hb is necessary and sufficient to specify early-born neuronal identity in multiple lineages, and is maintained in the post-mitotic neurons produced during each neuroblast expression window. Surprisingly, nothing is currently known about whether Hb acts in neuroblasts or post-mitotic neurons (or both) to specify first-born neuronal identity.

**Methods:**

Here we selectively remove Hb from post-mitotic neurons, and assay the well-characterized NB7-1 and NB1-1 lineages for defects in neuronal identity and function.

**Results:**

We find that loss of Hb from embryonic and larval post-mitotic neurons does not affect neuronal identity. Furthermore, removing Hb from post-mitotic neurons throughout the entire CNS has no effect on larval locomotor velocity, a sensitive assay for motor neuron and pre-motor neuron function.

**Conclusions:**

We conclude that Hb functions in progenitors (neuroblasts/GMCs) to establish heritable neuronal identity that is maintained by a Hb-independent mechanism.

We suggest that Hb acts in neuroblasts to establish an epigenetic state that is permanently maintained in early-born neurons.

**Electronic supplementary material:**

The online version of this article (doi:10.1186/s13064-017-0078-1) contains supplementary material, which is available to authorized users.

## Background

Development of the central nervous system (CNS) is multi-step process. In both mammals and *Drosophila*, the earliest steps are spatial patterning to define a neuroectodermal territory, followed by more precise spatial patterning to generate distinct progenitor domains (reviewed in [23, 43]). Subsequently, both mammals and *Drosophila* progenitors can sequentially express temporal transcription factors that specify neural identity based on birth-order (mouse: [2, 14, 32]) (fly: [3–7, 9, 18, 22, 24, 25, 34, 35, 37, 46]). The integration of spatial and temporal cues results in the production of a specific neuronal identity (reviewed in [1, 15, 38, 40]). Lastly, terminal selector genes regulate gene expression conferring distinct neural subtypes (e.g. dopaminergic neurons or cholinergic neurons); the defining feature of terminal selector genes is that their expression is maintained for the life of the neuron where it maintains the functional properties of the neuron (reviewed in [1, 21]).

**Fig. 1:**
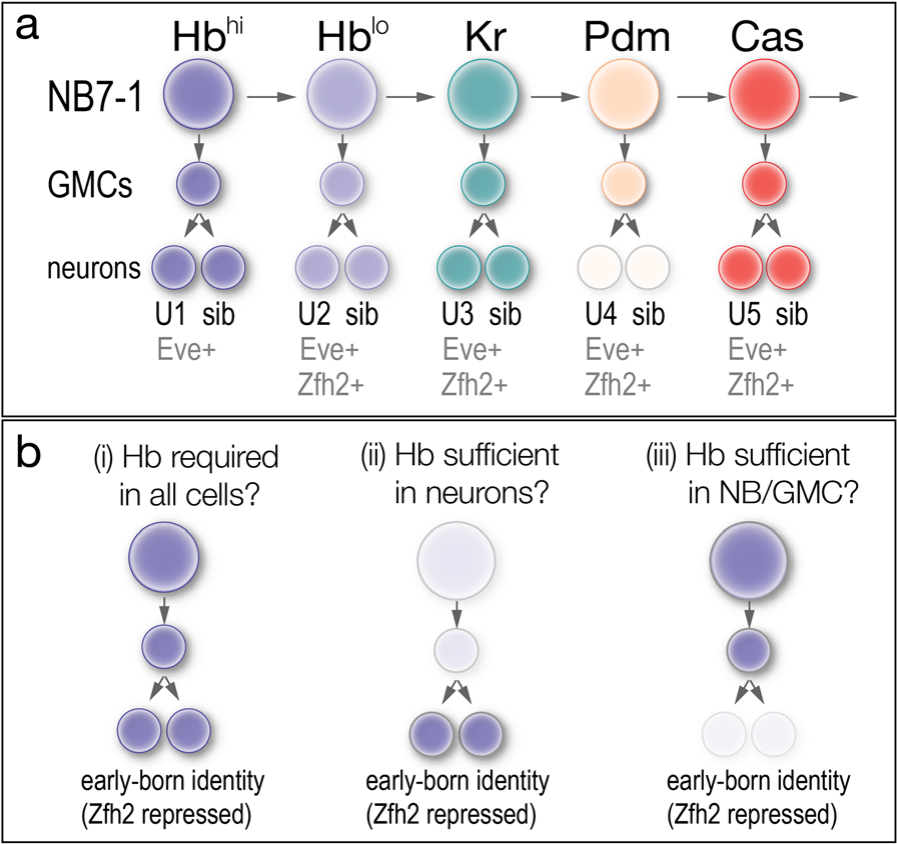
The NB7-1 lineage, temporal transcription factors, and models for Hunchback function in specifying early-born neuronal identity. **a** The neuroblast 7–1 (NB7-1) sequentially expresses the temporal transcription factors Hunchback (Hb), Kruppel (Kr), Pou domain proteins Nubbin/Pdm2 (Pdm), and Castor (Cas). Each factor is maintained into the post-mitotic neurons, although Pdm neuronal expression is transient. The U1-U5 motor neurons are Eve+; all but the first-born U1 express the late-born neuronal marker Zfh2. **b** Models for Hb specification of early-born neuronal identity. (i) Hb may be required in the neuroblast, GMC, and post-mitotic neurons, matching its expression pattern. (ii) Hb expression in post-mitotic neurons may be sufficient to maintain their identity; in this case the transient neuroblast expression may be a mechanism to limit Hb to early-born neurons. (iii) Hb expression in neuroblasts may be sufficient to heritably specify identity of their neuronal progeny, perhaps by initiating a transcriptional cascade or creating epigenetic modifications

Terminal selector genes are not the only class of regulators that are maintained by post-mitotic neurons. Many temporal transcription factors are not only transiently expressed during progenitor lineages, but also maintained in post-mitotic neurons produced during each progenitor expression window. For example, embryonic ventral nerve cord neuroblasts sequentially express the temporal transcription factors Hunchback (Hb), Kruppel, Pdm1/2 (Nubbin and Pdm2, Flybase), and Castor (reviewed in [38]); three of these factors -- Hb, Kruppel, and Castor -- maintain expression into post-mitotic neurons born during each neuroblast expression window (Fig. 1a). Similarly, optic lobe neuroblasts sequentially express Homothorax, Eyeless, Sloppy paired, Dichaete, and Tailless; the four earlier temporal stages generate neurons that inherit and maintain the temporal transcription factor present at their birth, although some Eyeless, Sloppy paired or Dichaete progeny lose temporal transcription factor expression [31]. Surprisingly, however, the role of temporal transcription factors in post-mitotic neurons has yet to be investigated.

Here we determine the role of the Hb temporal transcription factor in post-mitotic neurons of the well-characterized NB7-1 lineage [12, 17, 18, 22, 25, 28, 29, 33, 35, 37], with additional analysis of the role of Hb in post-mitotic neurons of the NB1-1 lineage [22]. The NB7-1 lineage is shown in Fig. 1a: it produces five GMCs that each express the homeodomain protein Even-skipped (Eve) just before they divide to produce the Eve+ U1-U5 motor neurons and their Eve- sibling interneurons [12, 37]. U1 maintains high Hb protein levels and represses Zfh2 expression; U2 maintains lower Hb protein levels and has low Zfh2 levels; U3-U5 have no Hb protein and have high Zfh2 levels [22]. Similar Eve, Hb, and Zfh2 expression patterns are observed for the NB1-1 and NB4-2 lineages [22]. Thus, Zfh2 expression can be used to distinguish first-born from later-born neurons in multiple neuroblast lineages. In addition, the first-born neurons in all three lineages have axon projections to dorsal body wall muscles, whereas later-born neurons project to more ventral muscles or are interneurons [22, 30].

The role of Hb in the NB7-1, NB1-1, and NB4-2 lineages has been well characterized. Loss of Hb throughout these lineages leads to failure to produce Eve+ early-born neurons (NB7-1) or failure to repress the late-born marker Zfh2 (NB1-1 and NB4-2). Conversely, misexpression of Hb throughout these lineages leads to ectopic Zfh2- neurons with axons targeting dorsal muscles similar to endogenous first-born neurons [22, 37]. Thus, Hb represses Zfh2 and specifies early-born neuronal identity in multiple neuroblast lineages. Hb is expressed in neuroblasts, GMCs, and neurons – which of these cell types requires Hb to establish or maintain early-born neuronal identity? Hb could be required in all cells (Fig. 1b, i); Hb could be required only in post-mitotic neurons (its transient expression in neuroblasts could be simply a mechanism to restrict its expression to early-born neurons; Fig. 1b, ii) or Hb could be required only in neuroblasts/GMCs (Fig. 1b, iii). Here we use cell type specific *hb* RNAi to selectively remove Hb from post-mitotic neurons, and show that Hb in neuroblasts/GMCs is sufficient for stable and long-lasting early-born neuronal identity (even when the neurons lack Hb protein). This is the first analysis of any *Drosophila* temporal transcription factor function in post-mitotic neurons, and it shows that the Hb temporal transcription factor functions differently from terminal selector genes, which are required to maintain post-mitotic neuronal properties (reviewed in [1, 21]).

## Methods

### Fly stocks

*en-gal4* – Bloomington stock 1973. Expressed in row 6/7 neuroectoderm and neuroblasts (A. Brand and K. Yoffe, unpublished) [22].

*CQ2-gal4 (II)* – Bloomington stock 7468. Expressed just prior to U1 birth.

*elav-gal4* – Bloomington stock 8760. Expressed all neuroblasts and neurons.

*UAS-hb RNAi* – Bloomington stock 34704. *hunchback* RNAi transgene.

*yellow white* (*yw*) – Bloomington stock 6598. Control for cell fate assays.

*UAS-mCherry RNAi* – Bloomington stock 35785. Control for behavior.

*hb* ^*P1*^, *hb* ^*FB*^/TM3 *ftzlacZ* – used to specifically remove *hb* CNS expression; *hb* ^*P1*^ is a transgene that recapitulates blastoderm *hb* expression; *hb* ^*FB*^ is a *hb* null allele [22].

*sca-gal4, UAS-hb* – used to express Hb in the neuroectoderm and neuroblasts [12, 37].

### Immunostaining, Imaging, and Figure preparation

Antibody staining was performed according to standard methods [28]. Primary antibodies, dilutions and sources were: chicken anti-GFP 1:500 (Aves Labs, Inc., Tigard, OR USA); rabbit anti-Hb 1:200 for embryos, 1:400 for larvae [46]; mouse anti-Even-skipped (3C10-c for embryos with 1:50, 2B8 for larvae with 1:75) and mouse anti-Engrailed (4D9) 1:5 (Developmental Studies Hybridoma Bank, University of Iowa, IA, USA); rat anti-Zfh2 1:500 [47]. Donkey anti-chicken Alexa Fluor 488-, donkey anti-rat Alexa Fluor 488-, donkey anti-rabbit Alexa Fluor 488-, donkey anti-mouse Alexa Fluor 647-, and donkey anti-rabbit Alexa Fluor 647-conjugated secondary antibodies were from Jackson ImmunoResearch (West Grove, PA USA). Goat anti-rabbit Alexa Fluor 555-, goat anti-mouse Alexa Fluor 555- and goat anti-rat Alexa Fluor 555-conjugated secondary antibodies were from Invitrogen (Eugene, OR USA). Confocal image stacks were collected using Zeiss LSM 700 confocal microscope, processed using ImageJ (NIH) and Photoshop (Adobe Systems Inc., Mountain View, CA USA); in some cases images were brightened using linear gain adjustment using the Levels command in Photoshop; when used, the entire panel was processed identically. Figures were assembled in Illustrator (Adobe Systems Inc., Mountain View, CA USA).

### Larval locomotor assays

Bright-field whole larval behavioral recordings were done using newly hatched first instar larvae. Behavior arenas were made of 6% agar in grape juice, 2 mm thick and 5.5 cm in diameter. The arenas were placed under a Leica S8APO dissecting microscope and red light (700 nm, Metaphase Technologies) illuminated a single larva. The microscope was equipped with a Scion1394 monochrome CCD Camera, using Scion VisiCapture software in ImageJ. Larva were tracked using FIM Tracker software [39]. For each larva, speed was calculated by dividing total distance traveled by the larval centroid by the time elapsed during the recording period.

## Results

### Loss of Hunchback from both neuroblast and neurons eliminates early-born neuronal identity

**Fig. 2:**
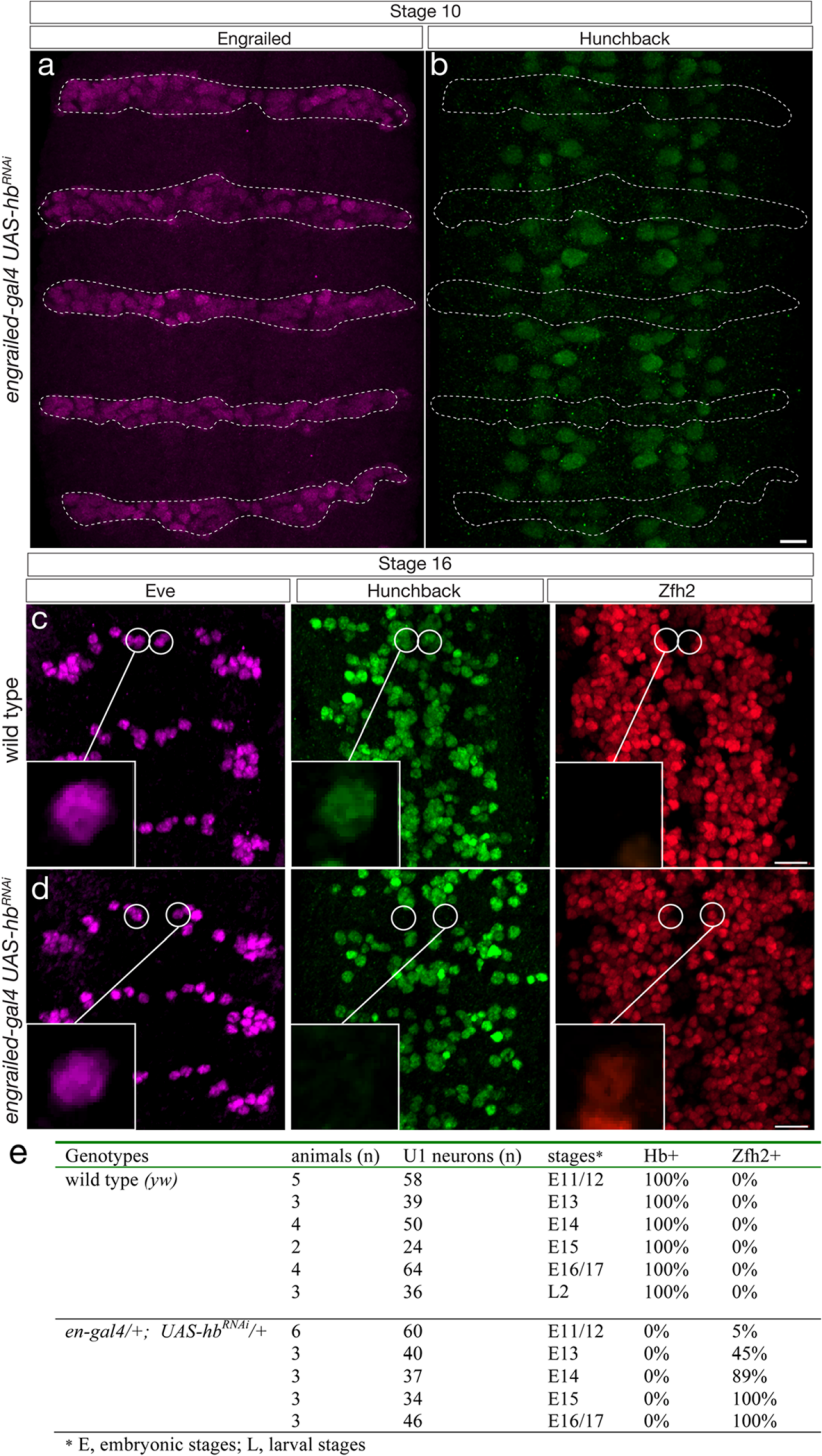
Loss of Hunchback from neuroblasts and neurons eliminates early-born neuronal identity. **a**, **b** Using *engrailed-gal4* (*en-gal4*) to drive *UAS-hunchback RNAi* (*UAS-hb* ^*RNAi*^) results in reduced Hb protein levels in row 6 and row 7 neuroblasts. Scale bars, 10 μm. **c** Wild type (*y w*) stage 16 embryo, three segments shown. U1 motor neurons (*circled*) are Eve+ Hb+ and Zfh2-. **d** *en-gal4 UAS-hb* ^*RNAi*^ stage 16 embryo, three segments shown. U1 motor neurons (*circled*) are Eve+ Hb- and Zfh2+. Anterior up, ventral midline at center of each panel, scale bar, 10 μm. **e** Quantification

We wanted to determine whether our *hb* RNAi transgene was strong enough to eliminate detectable Hb protein and replicate the *hb* null mutant phenotype. We expressed *UAS-hb* ^*RNAi*^ using *engrailed-gal4* (*en-gal4*), which is expressed in the posterior compartment neuroectoderm, NB7-1, and its U1-U5 neuronal progeny [18] (Additional file 1: Figure S1a). In wild type stage 10 embryos, all neuroblasts have uniform Hb expression [17, 22]. In contrast, *en-gal4 UAS-hb* ^*RNAi*^ stage 10 embryos have reduced Hb protein levels in neuroblasts within the *en-gal4 UAS-hb* ^*RNAi*^ domain (Fig. 2a,b).

We next determined whether *en-gal4 UAS-hb* ^*RNAi*^ could mimic the *hb* mutant phenotype in the NB7-1 lineage. In wild type stage 16 embryos, the Eve+ U1 motor neuron is Hb+ Zfh2- due to Hb repression of *zfh2* (Fig. 2c, quantified in 2e). In *en-gal4 UAS-hb* ^*RNAi*^ stage 16 embryos, the Eve+ U1 motor neuron lacks all detectable Hb protein and has de-repressed Zfh2 (Fig. 2d, quantified in 2e). This is a slightly weaker phenotype than genetic ablation of Hb expression in the NB7-1 lineage [22], perhaps due to the low levels of Hb in the neuroblast (see Discussion). We examined earlier stage embryos to determine when Hb was lost from the U1 neuron, and found that Hb is undetectable from the time of U1 birth at stage 11, although it takes several hours for Zfh2 to become de-repressed (Fig. 2e). We conclude that *hb* RNAi is capable of removing some Hb protein from NB7-1 and all detectable Hb protein from the U1 neuron, resulting in abnormal U1 neuronal identity.

### Loss of Hunchback from neurons does not alter early-born neuronal identity

**Fig. 3:**
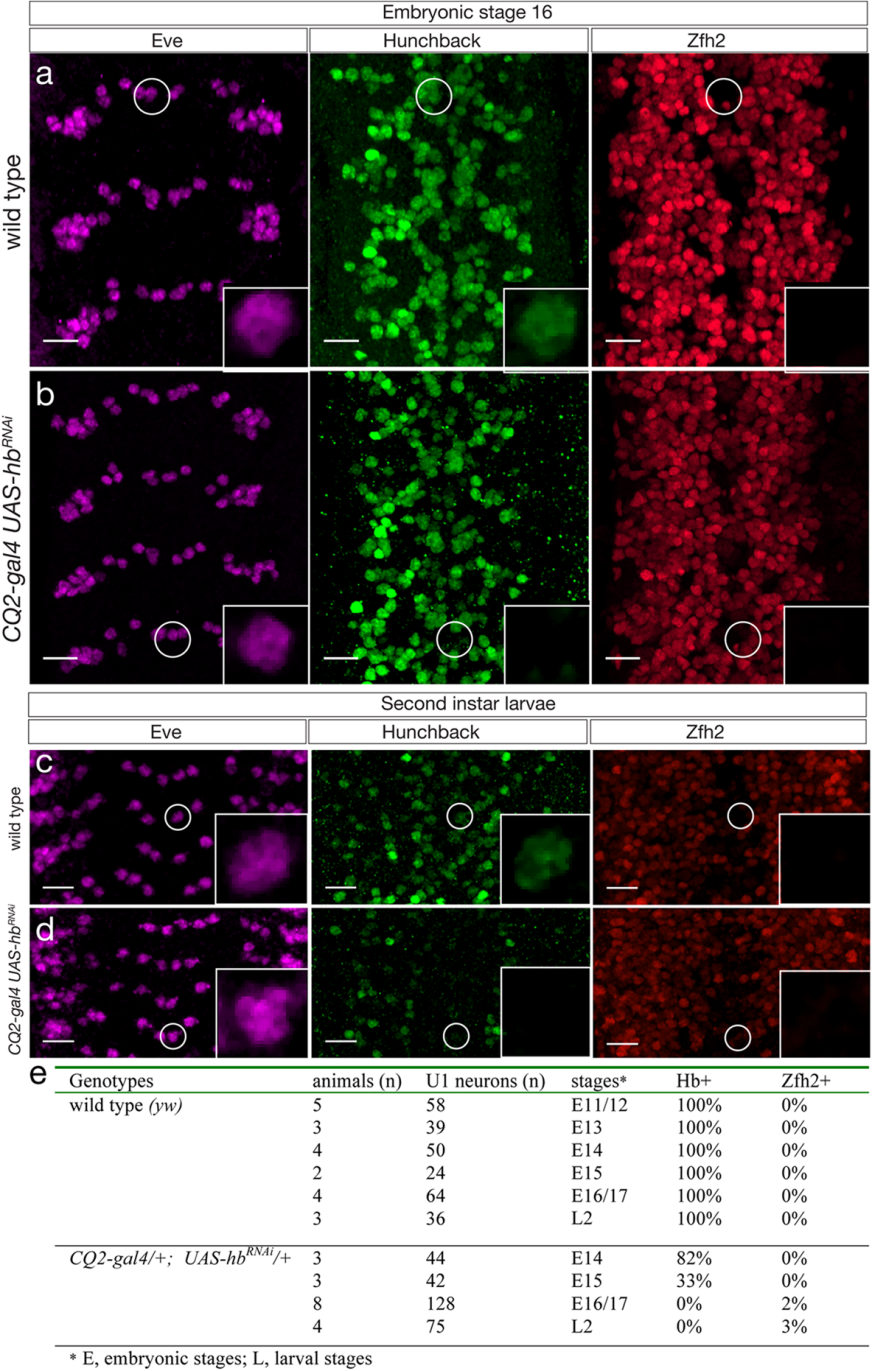
Loss of Hunchback from post-mitotic neurons does not alter early-born neuronal identity. **a** Wild type (*y w*) stage 16 embryo, three segments shown. The U1 neuron is Eve+ Hb+ Zfh2-; one example is circled and enlarged in the inset. **b** *CQ2-gal4 UAS-hb* ^*RNAi*^ stage 16 embryo, four segments shown. The U1 neuron is Eve+ Hb- Zfh2-; one example is circled and enlarged in the inset. Note that loss of neuronal Hb does not result in Zfh2 de-repression. **c** Wild type (*y w*) second instar larval CNS, four segments shown. The U1 neuron is Eve+ Hb+ Zfh2-; one example is circled and enlarged in the inset. **d** *CQ2-gal4 UAS-hb* ^*RNAi*^ second instar larval CNS, four segments shown. The U1 neuron is Eve+ Hb- Zfh2-; one example is circled and enlarged in the inset. Note that loss of neuronal Hb does not result in Zfh2 de-repression. Anterior up, ventral midline at center of each panel, scale bar, 10 μm. **e** Quantification

To determine if loss of Hb from the neuroblast or neuron leads to de-repression of Zfh2 and abnormal neuronal identity, we specifically decreased Hb from the U1 post-mitotic neuron. We used the *CQ2-gal4* (also called *eve-gal4* [12] or *eve+ 3.5-4.3-gal4* [37]) which has no NB7-1 expression but is expressed in GMCs about an hour before they divide to produce U motor neurons [12, 37] (Additional file 1: Figure S1b). In wild type stage 16 embryos, the Eve+ U1 motor neuron is Hb+ Zfh2- (Fig. 3a). In *CQ2-gal4 UAS-hb* ^*RNAi*^ stage 16 embryos, the Eve+ U1 motor neuron lacks all detectable Hb protein, but most have not de-repressed *zfh2* (Fig. 3b, quantified in 3e). These results show that Hb is not continuously required in the U1 neuron to maintain *zfh2* repression; this feature of early-born neurons must be established by transient Hb function in the neuroblast, GMC, or young neuron and heritably maintained by a Hb-independent mechanism.

To determine how long *CQ2-gal4 UAS-hb* ^*RNAi*^ can keep Hb levels off in the U1 motor neuron, and how long Zfh2 remains repressed in the absence of Hb, we examined *CQ2-gal4 UAS-hb* ^*RNAi*^ animals at the second larval instar stage. In wild type larvae, the Eve+ U1 neuron is Hb+ Zfh2- (Fig. 3c, quantified in 3e). In *CQ2-gal4 UAS-hb* ^*RNAi*^ larvae, the Eve+ U1 neuron is Hb- yet Zfh2 is still repressed (Fig. 3d, quantified in 3e). Thus, even prolonged loss of Hb does not lead to *zfh2* de-repression.

**Fig. 4:**
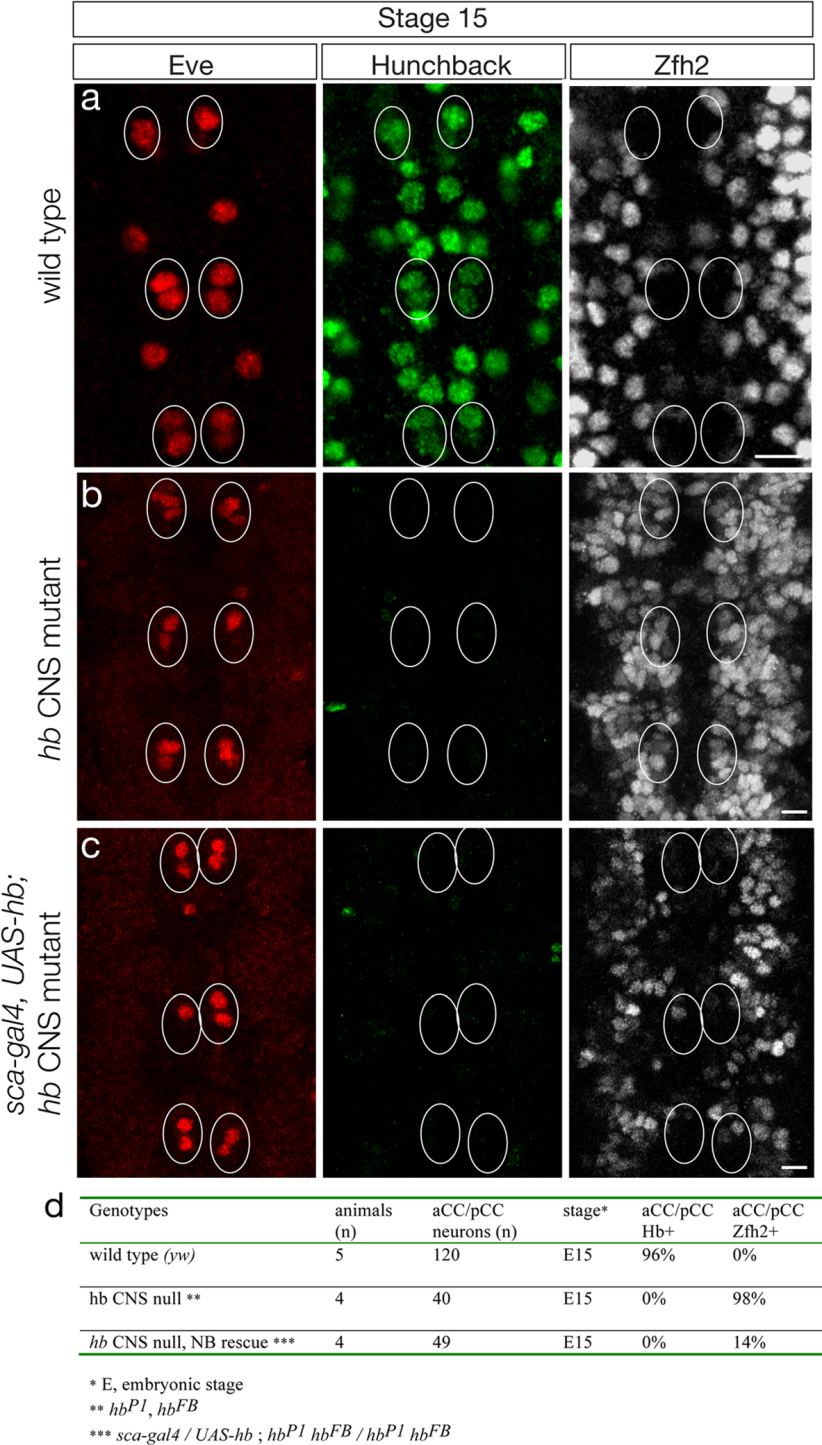
Transient Hunchback in neuroblasts is sufficient to specify early-born neuronal identity. **a** Wild type (*y w*) stage aCC/pCC sibling neurons (*circle*) are Eve+ Hb+ and Zfh2-. **b** *hunchback* CNS null mutant aCC/pCC sibling neurons (*circle*) are Eve+ Hb- and Zfh2+. Note that loss of Hb throughout the neuroblast lineage results in *zfh2* de-repression. **c** *hunchback* CNS null mutant with *sca-gal4 UAS-hb* driving expression of Hb in neuroblasts*.* aCC/pCC sibling neurons (*circle*) are Eve+ Hb- and Zfh2-. The Hb protein made in the parental neuroblast is not maintained in the aCC/pCC neuronal progeny. Note that Hb expression in the neuroblast maintains *zfh2* repression despite lack of Hb in the neurons. In all panels, stage 15 embryo, three segments shown, anterior to top, ventral midline at center of panel. Scale bars, 5 μm. **d** Quantification

We next used a completely independent method for neuron-specific elimination of Hb and assayed a second neuroblast lineage (NB1-1). We used a genotype lacking all Hb expression in the CNS (*hb* ^*P1*^ *hb* ^*FB*^ */hb* ^*P1*^ *hb* ^*FB*^; [22]) plus *sca-gal4 UAS-hb* transgenes that transiently drive Hb expression in neuroblasts, GMCs and young neurons [37] (Additional file 1: Figure S1c). In this experiment, we assayed the first-born Eve+ aCC/pCC sibling neurons from the NB1-1 lineage. In wild type stage 15 embryos, the Eve+ aCC/pCC neurons are Hb+ and repress the late-born marker Zfh2 (Fig. 4a, quantified in 4d). In *hb* CNS null mutants at stage 15, the Eve+ aCC/pCC neurons are Hb- and strongly de-repress Zfh2 (Fig. 4b, quantified in 4d)[22]. In stage 15 *hb* CNS null mutants that were transiently rescued with ectopic Hb earlier in the lineage, the Eve+ aCC/pCC neurons maintain repression of *zfh2 despite no longer having detectable Hb protein* (Fig. 4c, quantified in 4d). We propose that the transient expression of Hb in the NB1-1 lineage leads to epigenetic silencing of the *zfh2* locus, such that absence of Hb in older neurons does not lead to de-repression of *zfh2*. Supporting this conclusion is our previous observation that forced expression of Hb in late-born neurons (that normally are Zfh2+) does not lead to *zfh2* repression despite high levels of neuronal Hb protein [29, 37]. Taking all experiments together, we conclude that loss of Hb from three different post-mitotic neurons (U1, aCC, pCC) from two different neuroblast lineages (NB7-1, NB1-1) has no effect on the molecular identity of these early-born neurons: all are able to maintain repression of the late-born neuronal marker Zfh2 in the absence of Hb protein. We propose that Hb acts in the neuroblast, GMC or young neuron to heritably silence *zfh2* expression (see Discussion).

### Loss of Hunchback from neurons only does not alter early-born neuronal morphology

To determine if loss of Hb from the post-mitotic U1 neuron leads to alteration in neuronal morphology, we used *CQ2-gal4* to drive expression of *UAS-hb* ^*RNAi*^ (to remove Hb protein) and *UAS-myristoylated (myr):GFP* (to reveal U1-U5 neuronal morphology). Although myr:GFP was expressed in most or all U1-U5 motor neurons, only the U1 motor neuron projects to the most dorsal muscles of the body wall [22, 30], which allows us to detect any defects in U1 morphology.

**Fig. 5:**
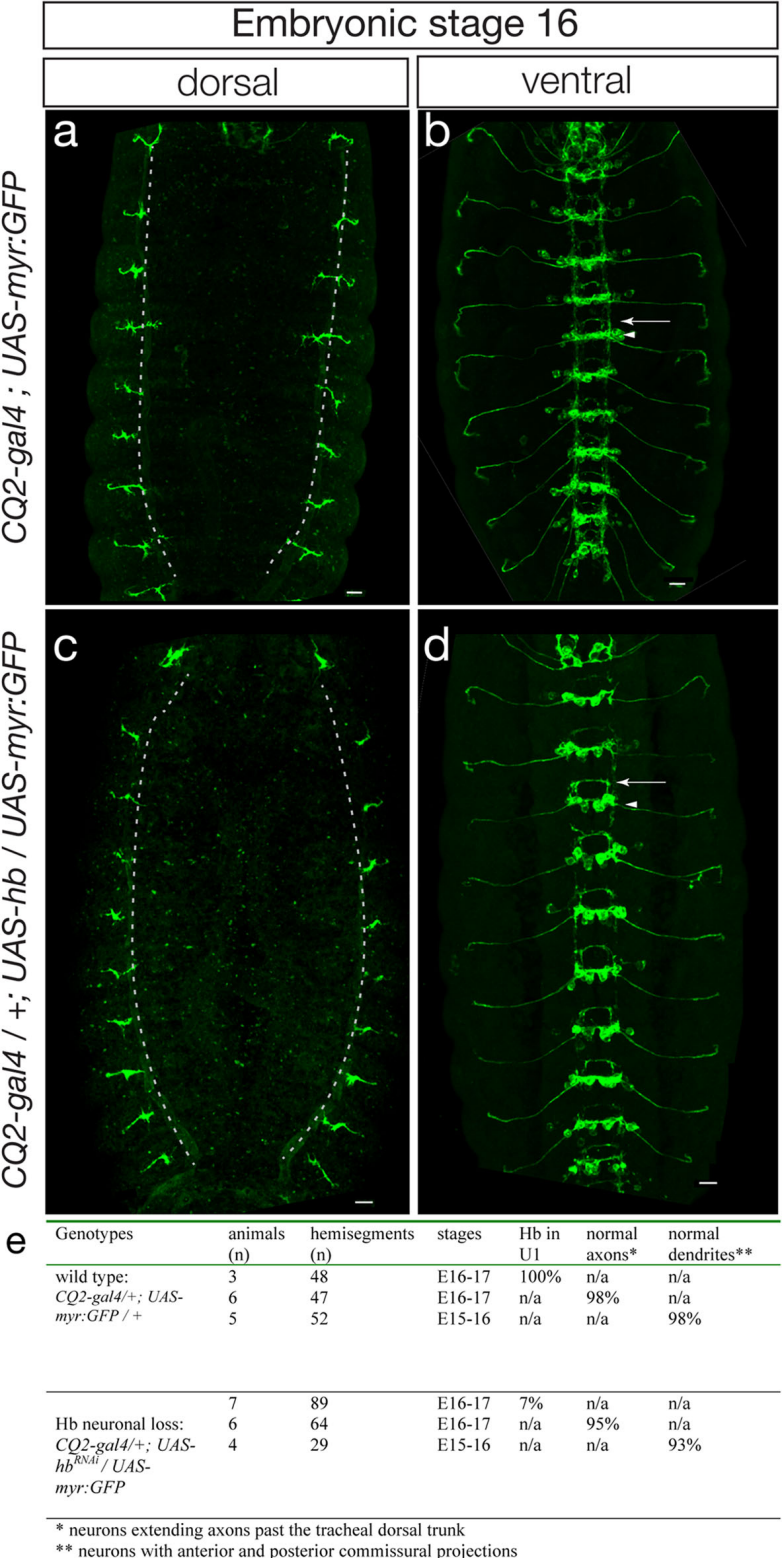
Loss of Hunchback from post-mitotic neurons only does not alter embryonic neuronal morphology. U1-U5 motor neuron morphology detected by *CQ2-gal4* driving expression of *UAS-myristoylated:GFP* (*green*). Only the Hb+ U1 and U2 motor neurons project to the most dorsal muscles [22, 30]. **a**, **b** Wild type *CQ2-gal4;UAS-myr:GFP* stage 16 embryo. **a** The most dorsal projecting U1/U2 motor neurons extend past the tracheal dorsal trunk (*dashed lines*). **b** Dendritic projections form a thick posterior commissural fascicle (*arrow*) and a thin anterior commissural fascicle (*arrowhead*) in each segment; note faint processes in most intersegmental connectives. **c**, **d** *CQ2-gal4/+; UAS-hb* ^*RNAi*^/*UAS-myr:GFP* stage 16 embryo. **c** The most dorsal projecting U1/U2 motor neurons extend past the tracheal dorsal trunk (*dashed lines*). No difference is seen in axon or dendrite morphology between wild type and *CQ2-gal4 UAS-hb* ^*RNAi*^ embryos. **d** Dendritic projections form a thick posterior commissural fascicle (*arrowhead*) and a thin anterior commissural fascicle (*arrow*) in each segment; note slightly reduced processes in most intersegmental connectives. Anterior up, dorsal (**a**,**c**) or ventral (**b**,**d**) midline at center of each panel, scale bar, 10 μm. **e** Quantification

In wild type stage 16 embryos, the U1 motor neuron projected to the most dorsal muscles, past the dorsal branch of the trachea (Fig. 5a, quantified in 5e). In *CQ2-gal4 UAS-hb* ^*RNAi*^ stage 16 embryos, the U1 motor neuron also projected past the dorsal branch of the trachea (Fig. 5c, quantified in 5e). Although we can't distinguish the U1 dendrites from the U2-U5 dendrites, we saw only minor, transient differences in dendritic projections within the CNS. For example, projections in the connectives were typically slightly weaker in hb RNAi embryos compared to control embryos (Fig. 5b, d), although by second instar larvae we see no difference in the projections within the connectives (Additional file 2: Figure S2). We conclude that loss of Hb from the post-mitotic U1 motor neuron does not alter its neuronal morphology.

### Loss of Hunchback from U1/U2 neurons or all neurons does not alter larval locomotor velocity

**Fig. 6:**
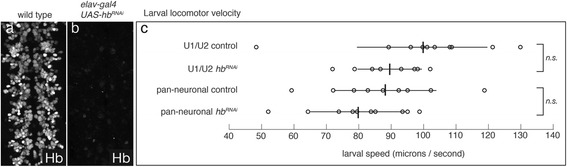
Loss of Hunchback from U1/U2 motor neurons or all neurons does not alter larval locomotor velocity. **a** Wild type *yw* stage 16 embryo stained for Hb protein. **b** *elav-gal4 UAS-hb* ^*RNAi*^ stage 16 embryo stained for Hb protein. **c** Loss of Hb from the U1/U2 motor neurons or all neurons does not alter larval locomotor velocity. Genotypes: U1/U2 control = *CQ2-gal4 UAS-mCherry* ^*RNAi*^. U1/U2 *hunchback* RNAi = *CQ2-gal4 UAS-hb* ^*RNAi*^. Pan-neuronal control = *elav-gal4 UAS-mCherry* ^*RNAi*^. Pan-neuronal *hunchback* RNAi = *elav-gal4 UAS-hb* ^*RNAi*^. Average speed for each genotype (*vertical line*) and standard deviation (*horizontal line*) are shown overlaid on distance/time (n is 1–2 crawls for 5 larva of each genotype) (*circles*)

We have previously characterized the role of motor neurons and interneurons in larval locomotion, and have developed a method to quantify larval locomotor velocity, a sensitive read-out for normal motor neuron function [11, 19, 20]. It is possible that loss of Hb from the U1 and U2 motor neurons (or all early-born neurons) may not alter gene expression or neuronal morphology, but rather affect motor neuron function leading to locomotor defects. To determine if loss of Hb from the post-mitotic U1/U2 neurons leads to defects in larval locomotor velocity, we used *CQ2-gal4* to drive expression of *UAS-hb* ^*RNAi*^. We found that *CQ2-gal4 UAS-hb* ^*RNAi*^ removed all detectable Hb protein from the U1 motor neuron (see Fig. 3d) but had no significant effect on larval locomotor velocity (Fig. 6c, top two rows). We also used *elav-gal4* to drive *UAS-hb* ^*RNAi*^ to remove Hb from all post-mitotic neurons and assay for defects in larval locomotor velocity. Note that although *elav-gal4* is expressed in neuroblasts, this expression begins at stage 11–12 [8, 26], after Hb expression normally fades from neuroblasts, and has no effect on Hb expression in neuroblasts (data not shown). Thus, *elav-gal4 UAS-hb* ^*RNAi*^ selectively removes Hb from all neurons without affecting neuroblast/GMC expression. Although *elav-gal4 UAS-hb* ^*RNAi*^ animals lack virtually all neuronal Hb protein (Fig. 6a,b), we observed no significant effect on larval locomotor velocity (Fig. 6c, bottom two rows). The fact that widespread loss of Hb from early-born motor neurons and interneurons has no effect on larval locomotor velocity provides strong evidence that Hb is not required to maintain functional properties of mature neurons.

## Discussion

Here we show that the temporal transcription factor Hb, despite being continuously expressed in the U1 motor neuron, is not required to maintain U1 neuronal identity. We conclude that Hb acts transiently in the neuroblast, GMC, or new-born neuron to establish the U1 neuronal identity, and that this identity is subsequently maintained by a Hb-independent mechanism. Our conclusion is also supported by the observation that Hb, like many temporal transcription factors, are re-used in other cell types or tissues to specify different cell fates, showing that cellular context shapes the response to Hb. It is likely that progenitors and post-mitotic neurons provide different contexts for Hb action; the role of Hb in early-born post-mitotic neurons has yet to be defined. For example, in the neuroblast/GMC progenitors, Hb confers temporal identity [22], in the early embryo Hb specifies anterior-posterior identity [36], and in adult male neurons Hb confers male-specific morphology [16]. Similar findings are observed for other embryonic temporal transcription factors such as Kr, Pdm, and Castor [10, 22, 42, 44, 46].

Interestingly, our attempts to remove Hb from the entire NB7-1 lineage using *en-gal4 UAS-hbRNAi* resulted in residual Hb protein in NB7-1 and a weaker phenotype than complete genetic removal of Hb from the NB7-1 lineage [22]. For example, both *hb* RNAi and *hb* null mutants resulted in U1 motor neurons that de-repressed *zfh2* ([22]; Fig. 2) but only genetic *hb* null mutants result in absence of early-born Eve+ neurons ([22]; Fig. 2). This suggests that the Hb protein present in the NB7-1 following *hb* RNAi is sufficient to produce long-lasting expression of Eve in the U1 motor neuron.

We conclude that Hb has no detectable function in post-mitotic U1 neurons. Might this lack of phenotype be due to low levels of residual Hb protein in neurons? Although we can't formally rule this out, there are several reasons to discount this possibility. First, we stain for Hb protein and find most U1 neurons have no detectable Hb protein compared to background. Second, RNAi knockdown of Hb in neuroblasts produces a strong phenotype which would not be expected if very low levels of Hb are functional. Finally, we don't expect Hb protein to persist following loss of hb RNA, as we have previously shown that Hb protein in the CNS has a very short half life. These experiments co-stained neuroblasts and their progeny for Hb protein and active *hb* transcription (nuclear intron signal), and found that few or no cells had Hb protein but not *hb* transcription [17].

Our conclusion that Hb has no function in post-mitotic neurons is buttressed by our previous findings that late-born Hb-negative neurons are unaffected by forced Hb misexpression [12, 37]. We hypothesize that temporal transcription factors alter the epigenetic state of neuroblasts which is inherited by their progeny neurons. Thus, early-born neurons do not need Hb to maintain early-born identity, and are also unresponsive to forced expression of other temporal transcription factors; similarly, late-born neurons are unresponsive to forced expression of early temporal transcription factors [12, 37]. This model is supported by findings that Hb acts transiently at the cellular blastoderm stage together with the chromatin remodeler Mi-2 to permanently silence the *Ubx* gene [27]. It is also supported by the observation that some temporal transcription factors are only transiently expressed in progenitors and new-born neurons, such as Pdm in embryonic lineages [18, 22] or Eyeless, Sloppy paired, Dichaete, and Tailless in larval optic lobe lineages [31]. In these cases, the temporal transcription factor must act transiently in the neuroblast or GMC to confer long-lasting neuronal identity. Our findings raise the possibility that all temporal transcription factors are required transiently in progenitors to specify permanent temporal identity, despite many of these factors being maintained in post-mitotic neurons. If our findings can be extended to other temporal transcription factors, it would highlight the differences between spatial or temporal patterning genes (required transiently in progenitors) and terminal selector genes (required permanently in post-mitotic neurons). It would also highlight the importance of properly linking spatial/temporal patterning to terminal selector gene expression, an important area for future investigation.

We can’t rule out the possibility that Hb is required in post-mitotic neurons for aspects of neuronal function that we did not assay. In fact, post-embryonic expression of Hb is required for proper Fruitless + male neurons morphogenesis; following hb RNAi these neurons are transformed to a female-like morphology [16]. Although we did not detect striking axon or dendrite changes in the U neurons following hb RNAi, we did observe a slight decrease in neuronal projections in connectives (Fig. 5). Although Hb is not required to maintain dorsal axon projections in embryonic or larval U1 motor neurons, but it may be required for proper ion channel or neurotransmitter production. Furthermore, mammalian post-mitotic neurons can be reprogrammed to another neuronal identity for a short time after their birth [41]. Temporal transcription factors like Hb may stabilize neuronal identity to prevent such transformations; in this case, loss of neuronal Hb would only show a strong phenotype upon misexpression of a “reprogramming factor,” such as a later temporal transcription factor or a terminal selector gene for a different neural subtype.

Perhaps the strongest evidence we have against a Hb function in post-mitotic neurons is our finding that elimination of Hb protein from all post-mitotic neurons (*elav-gal4 UAS-hb* ^*RNAi*^, Fig. 6) has no larval locomotor phenotype. Similar experiments driving pan-neuronal expression of neuronal silencers or activators leads to larval paralysis [11, 13, 45]. Thus, it is highly unlikely that loss of Hb alters early-born interneuron or motor neuron neurotransmitter phenotypes or membrane properties. In the future, it would be interesting to use transcriptional profiling to compare Hb+ and Hb- early-born neurons– our results suggest that there would be little transcriptional effect from removing Hb from post-mitotic neurons.

## Conclusions

We conclude that the Hb functions in progenitors (neuroblasts/GMCs) to establish heritable neuronal identity that is maintained by a Hb-independent mechanism.

We suggest that Hb acts in neuroblasts to establish an epigenetic state that is permanently maintained in early-born neurons.

## Additional files

Additional file 1: Figure S1. Summary of three transgenic Gal4 line expression patterns in the NB7-1 or NB1-1 lineages. Black, high level; gray, low level; white, no expression. aCC, anterior corner cell; pCC, posterior corner cell. (TIF 1150 kb)
Additional file 2: Figure S2. Loss of Hunchback from post-mitotic neurons only does not alter embryonic neuronal morphology. U1-U5 motor neuron morphology detected by *CQ2-gal4* driving expression of *UAS-myristoylated:GFP* (green). (a) control *CQ2-gal4/+;UAS-myr:GFP/UAS-mCherry* ^*RNAi*^L2 larval CNS. Note projections out motor nerve roots and robust CNS projections. (b) *CQ2-gal4/+;UAS-hb* ^*RNAi*^/*UAS-myr:GFP* L2 larval CNS. Note projections out motor nerve roots and robust CNS projections. (TIF 3032 kb)

## Abbreviations

CNS: Central nervous system
Eve: Even-skipped
Hb: Hunchback
