# Supplementary figures and images for: The Hunchback temporal transcription factor establishes, but is not required to maintain, early-born neuronal identity
Source: Neural Dev. 2017 Jan 31;12:1. doi: 10.1186/s13064-017-0078-1 (PMC5282720; doi:10.1186/s13064-017-0078-1)

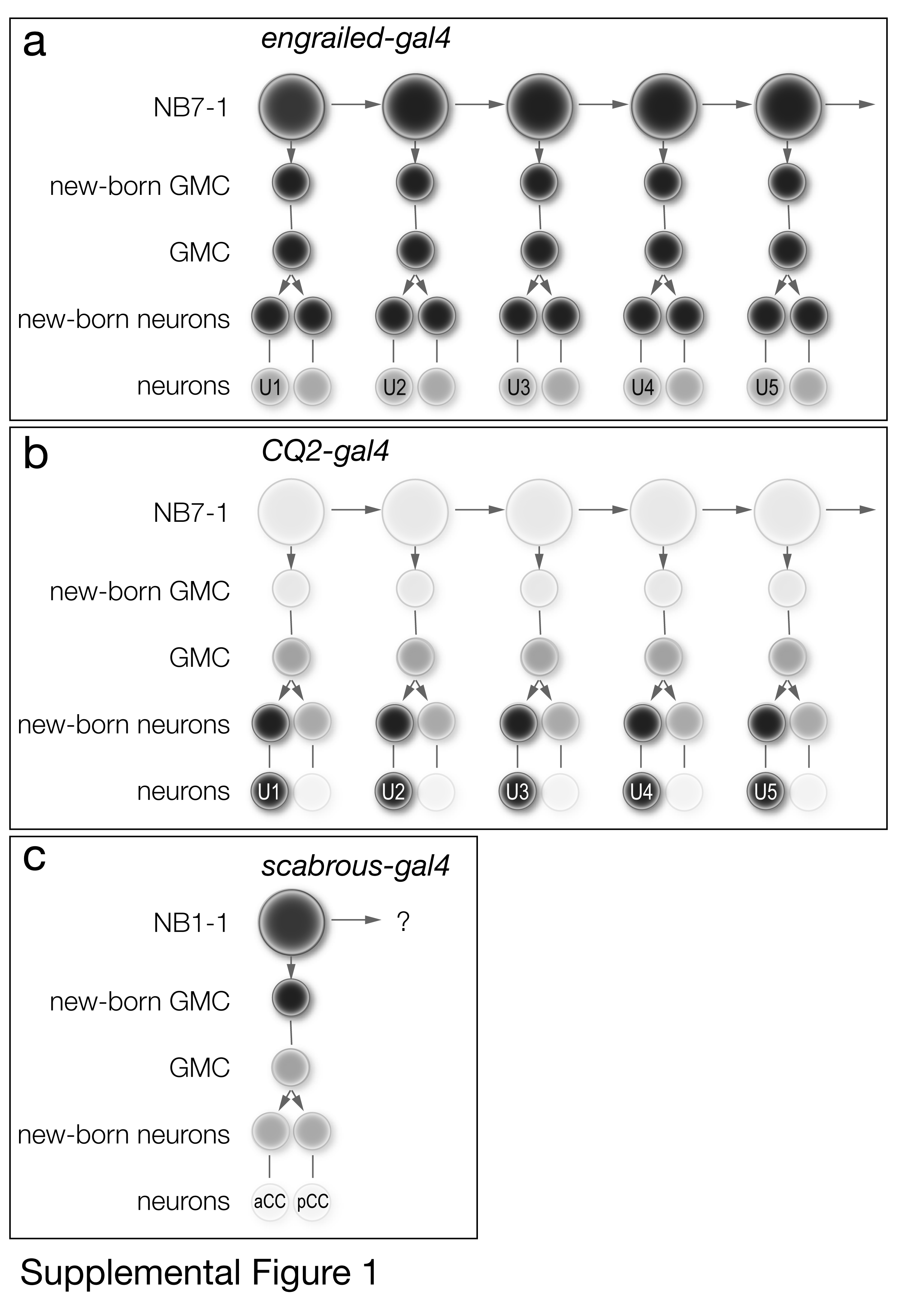

Supplement: Additional file 1: Figure S1. — Summary of three transgenic Gal4 line expression patterns in the NB7-1 or NB1-1 lineages. Black, high level; gray, low level; white, no expression. aCC, anterior corner cell; pCC, posterior corner cell. (TIF 1150 kb) [file 13064_2017_78_MOESM1_ESM.tif]

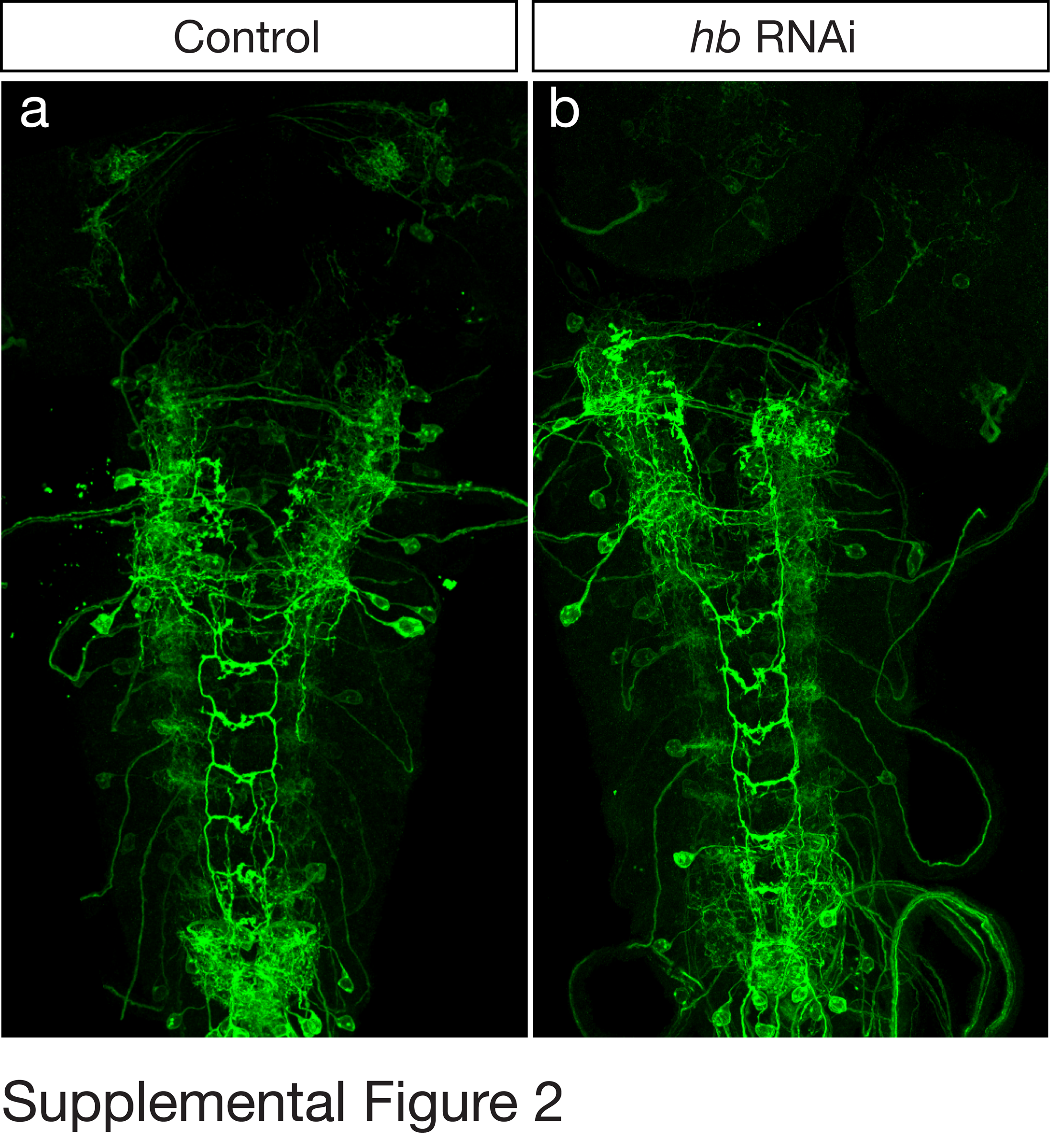

Supplement: Additional file 2: Figure S2. — Loss of Hunchback from post-mitotic neurons only does not alter embryonic neuronal morphology. U1-U5 motor neuron morphology detected by CQ2-gal4 driving expression of UAS-myristoylated:GFP (green). (a) control CQ2-gal4/+;UAS-myr:GFP/UAS-mCherry RNAiL2 larval CNS. Note projections out motor nerve roots and robust CNS projections. (b) CQ2-gal4/+;UAS-hb RNAi/UAS-myr:GFP L2 larval CNS. Note projections out motor nerve roots and robust CNS projections. (TIF 3032 kb) [file 13064_2017_78_MOESM2_ESM.tif]
